# Short-term outpatient follow-up of vericiguat treatment in patients hospitalized for heart failure

**DOI:** 10.3389/fcvm.2025.1465700

**Published:** 2025-03-04

**Authors:** Zihan Li, Tingting Li, Tingxun Liu, Yuanqiao Liu, Daoyuan Si, Yuquan He, Ping Yang

**Affiliations:** Department of Cardiology, Engineering Laboratory for Endothelial Function, China-Japan Union Hospital of Jilin University, Changchun, Jilin, China

**Keywords:** vericiguat, heart failure, heart failure hospitalization, safety, efficiency

## Abstract

**Background:**

Vericiguat—a novel oral soluble guanylate cyclase stimulator—was developed for the treatment of chronic heart failure (HF). Although the value of vericiguat therapy in chronic HF has been gradually recognized, its safety and efficacy in the acute phase of HF remain elusive.

**Methods:**

100 patients with acute HF receiving vericiguat therapy at the China-Japan Union Hospital of Jilin University between September 2022 and June 2023 were retrospectively analyzed. An external control was built from real-world data of acute HF subjects contemporaneously hospitalized in the same hospital using a propensity score matching (PSM) method.

**Results:**

After a median follow-up of 68 days, 80 patients completed at least one outpatient follow-up or had an endpoint event and cardiovascular death occurred in 6 patients. We matched 75 external control patients for this purpose. In single-arm study, overall, although systolic blood pressure (SBP) decreased significantly before and after treatment, there was little change in SBP in the SBP low group (baseline SBP less than 120mmHg) (from 109 mmHg to 105 mmHg, *p* = 0.109). Estimated glomerular filtration rate (eGFR) and serum potassium did not change significantly (*p* = 0.521 and 0.070, respectively). However, compared with the renal function normal group, eGFR showed a slower downward trend in the renal insufficiency group (*p* = 0.025). After using the PSM method, significant improvements in left ventricular ejection fraction and N-terminal pro-B-type natriuretic peptide were seen in both groups before and after treatment. There was no significant difference between the two groups. However, the downward trend in eGFR was even less significant in the vericiguat group, with significant differences between the two groups (*p* = 0.024).

**Conclusions:**

Vericiguat is feasible in acute HF, even in patients with hypotension and renal dysfunction. At the same time, vericiguat may have a potential renoprotective effect, which warrants further exploration.

## 1 Introduction

Heart failure (HF) is a severe manifestation or advanced stage of various heart diseases that affects approximately 64.3 million people worldwide (1). Despite advances in therapeutic drugs, HF still leads to high mortality and hospitalization rates. Therefore, the identification of new treatment strategies is urgent.

Vericiguat—as a novel oral soluble guanylate cyclase (sGC) stimulator—enhances the cyclic guanosine monophosphate (GMP) pathway by directly stimulating sGC through a binding site independent of nitric oxide (NO) and sensitizes sGC to endogenous NO by stabilizing NO binding to the binding site (2). Large phase III clinical trials showed that compared with placebo, vericiguat therapy reduced cardiovascular mortality and HF hospitalization in patients with stable heart failure with ejection fraction (HFrEF) accompanying a high risk of worsening HF (3). Some of the existing guidelines also acknowledge the important role of vericiguat in chronic HF (4, 5).

Based on our literature search, no clinical studies on the use of vericiguat in acute decompensated HF have been identified. Regardless of the type of HF, the safety and efficacy of vericiguat in the acute phase of HF remains uncertain. Herein, we evaluate the safety and efficacy of vericiguat therapy in decompensated HF and provide new evidence for its clinical application.

## 2 Materials and methods

### 2.1 Single-arm study

To evaluate the preliminary safety and efficacy of the vericiguat in acute phase of HF subjects, a single-arm investigator-initiated study was conducted at China-Japan Union Hospital of Jilin University between September 2022 and June 2023, in China. The enrolled patients had symptoms and signs of acute decompensated HF at admission, such as chest tightness, shortness of breath, dyspnea, and edema of both lower limbs. These HF patients contained a variety of categories, including HFrEF (*n* = 46), heart failure with mild reduced ejection fraction (HFmrEF) (*n* = 25) and heart failure with preserved ejection fraction (HFpEF) (*n* = 9). Patients with a first diagnosis of HF were included, besides, their common feature was that none of them had received guideline-directed medical therapy prior to this hospitalization. Patients who were lost to follow-up or did not receive vericiguat for more than one month were excluded.

All of them were treated with vericiguat before discharge and continued to outpatient follow-up. Baseline characteristics of the patients, including laboratory, echocardiographic, etiology, comorbidity, diagnosis and medication data, were collected. After a median follow-up of 68 days, a total of 20 patients were lost to follow-up. Among them, 10 patients refused outpatient follow-up due to their health status (7 patients felt they had recovered well, and 3 patients were bedridden at home due to severe illness); 6 patients refused outpatient follow-up due to time, financial, or other reasons; and 4 patients could not be contacted due to relocation, phone number changes, or death.

**Figure 1:**
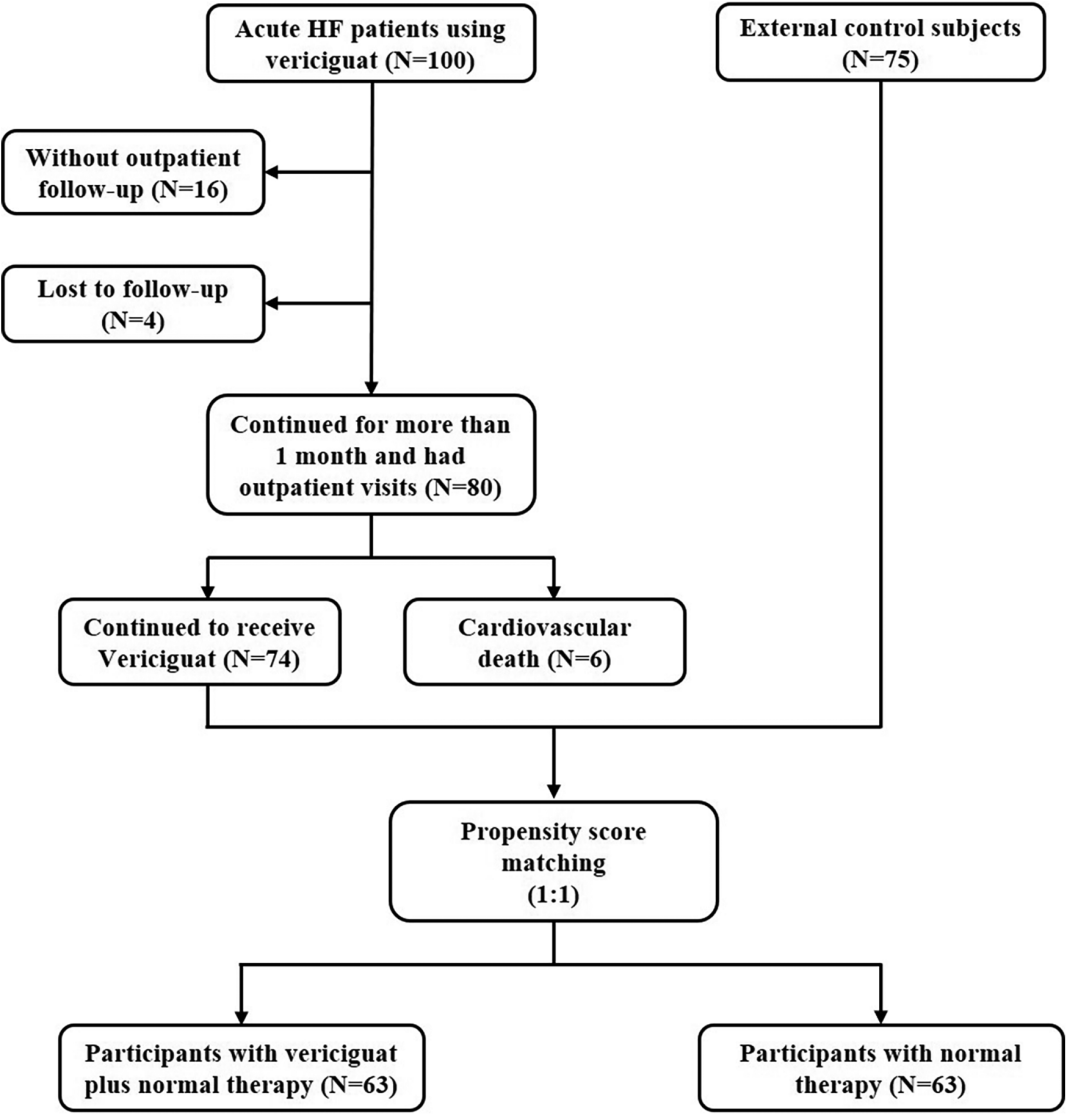
Patient selection in our study is shown.

Based on previous relevant clinical studies, we ultimately selected the following indicators as observation parameters and endpoint events (6–9). Observation indices for efficacy included left ventricular ejection fraction (LVEF), left ventricular diameter (LVD), left atrium diameter (LAD) and mitral regurgitation area (MRA) measured by echocardiography for outpatient follow-up, as well as the laboratory index N-Terminal Pro-Brain Natriuretic Peptide (NT-proBNP). Observation indices for safety included symptomatic hypotension, blood potassium and estimated glomerular filtration rate (eGFR). End events included all-cause death and cardiovascular death. Echocardiograms in this study were conducted by experienced sonographers who specialize in the heart. The LVEF values of each patient were measured three times and averaged to give a paper version of the report. A flow chart of patient selection is shown in Figure 1.

### 2.2 External control group and PSM method

Real-world data are collected from electronic health records of patients with acute decompensated HF admitted to the same hospital and used to form an external control group. These subjects received anti-HF drug therapy with the exception of Vericiguat only and were hospitalized during the same period as the single-arm study. Their individual patient-level data (IPD) were retrospectively analyzed by applying the inclusion/exclusion criteria of the single-arm study and using the propensity score matching (PSM) method to establish the external control group.

PSM was employed in this study due to the potential for confounding variables in case-control studies, which can lead to biased results. PSM is a statistical method used to handle research data by selecting and matching baseline characteristics of the groups to balance confounding variables, thereby minimizing bias in the study outcomes (10). PSM was performed using a multivariate logistic regression model to adjust for differences in baseline characteristics [age, gender, baseline LVEF (11, 12)] in the matched analysis. A propensity score was given for each subject in both the vericiguat group and the external control group. Subjects were then matched by propensity score in a 1:1 ratio using the caliper matching and a caliper size of 0.02. The standardized mean difference (SMD) for each covariate was calculated and computed to assess the balance in baseline characteristics before and after matching. The variables with a SMD < 0.1 are considered to be well-balanced between the vericiguat group and the external control group.

### 2.3 Oversight

The investigation conforms with the principles outlined in the Declaration of Helsinki. This trial was approved by the Ethical Review Board of China-Japan Union Hospital of Jilin University (approval No. 2023071116) and was registered in the Chinese Clinical Trial Registry (ChiCTR2300077292).

### 2.4 Statistical analysis

Continuous variables were presented as median (interquartile range), and normality tests were conducted. For normally distributed data with equal variances, *t*-tests were used; for data with unequal variances, Welch's *t*-test was applied. Non-normally distributed data were analyzed using the Mann–Whitney *U*-test. Categorical variables were expressed as frequency (percentage) and analyzed using the chi-square test or Fisher's exact test, as appropriate. Statistical analyses were performed using SPSS version 25.0 (IBM Corp). Variables with *p* < 0.05 were considered statistically significant.

## 3 Results

### 3.1 Baseline characteristics of single-arm study

A total of 100 patients with prescriptions for at least 30 days were included in the analysis. Among them, 16 patients did not complete the outpatient follow-up and 4 patients were lost to follow-up. Finally, only 80 patients had at least one month of follow-up echo examination data or endpoint. These patients included a first episode of acute decompensated heart failure as well as a second acute episode of admission after multiple previous episodes.

The baseline characteristics of the study population are displayed in Table 1. The median age was 63 (55, 70) years, with males accounting for 77.5% of the entire cohort. The most common etiology for HF was ischemic cardiomyopathy (82.5%), followed by dilated cardiomyopathy, hypertrophic cardiomyopathy, peripartum cardiomyopathy, etc. At baseline, the median creatinine level was 80.69 (64.88, 101.58) μmol/L and the median eGFR was 84.27 (67.57, 98.96) ml/min/1.73 m2. The median NT-proBNP level was 3,425 (1,223, 9,865) pg/ml. For echocardiography results, the median LVEF was 35.7 (29.0, 45.0) %, with a median LVD of 56.3 (49.0, 64.2) mm, a median LAD of 42.8 (37.7, 49.4) mm and a median MRA of 4.3 (1.8, 7.8) cm2 at baseline. Regarding medication, 66 (82.5%) patients received renin-angiotensin system inhibitors, 70 (87.5%) patients received b-blockers, 71 (88.7%) received mineralocorticoid receptor antagonists and 73 (91.2%) patients received sodium-dependent glucose transporters 2 inhibitors at baseline.

**Table 1:** Characteristics of the patients in single-arm study at baseline.

Parameter	The current study (*n* = 80)
Basic characteristics	
Age	63 (55, 70)
Male gender	62 (77.5%)
Systolic blood pressure	120 (108, 138)
Comorbidities	
Hypertension	38 (47.5%)
Diabetes mellitus	16 (20.0%)
Atrial fibrillation	8 (10.0%)
After coronary stent	19 (23.8%)
Etiology	
Ischemic cardiomyopathy	66 (82.5%)
Nonischemic cardiomyopathy	14 (17.5%)
Laboratory data	
Creatinine	80.69 (64.88, 101.58)
eGFR	84.27 (67.57, 98.96)
Potassium	4.3 (4.0, 4.6)
NT-proBNP	3,425 (1,223, 9,865)
Echocardiography	
Left ventricular ejection fraction	35.7 (29.0, 45.0)
Left atrium diameter	42.8 (37.7, 49.4)
Left ventricular diameter	56.3 (49.0, 64.2)
Mitral regurgitation area	4.3 (1.8, 7.8)
Medications	
β-blocker	70 (87.5%)
ACEI/ARB/ARNI	66 (82.5%)
MRA	71 (88.7%)
SGLT-2i	73 (91.2%)
Furosemide	50 (62.5%)
Tolvaptan	2 (2.5%)
Digoxin	6 (7.5%)

eGFR, estimated glomerular filtration rate; NT-proBNP, N-terminal pro-brain natriuretic peptide; ACEI, angiotensin converting enzyme inhibitor; ARB, angiotensin receptor blocker; ARNI, angiotensin receptor/neprilysin inhibitor; MRA, mineralcorticoid recept antagonist; SGLT-2i, sodium-glucose cotransporter protein-2 inhibitors.

### 3.2 Safety analysis

At the end of the study, 74 patients continued to receive vericiguat therapy, of which 13 (17.6%) patients received 10 mg, 43 (58.1%) received 5 mg and 18 (24.3%) received 2.5 mg of vericiguat.

The safety analysis is detailed in Table 2. Overall, systolic blood pressure (SBP) declined significantly from 120 (110, 139) mmHg to 110 (104, 130) mmHg (*p* < 0.001). Patients were divided into a systolic-blood-pressure low (SBPL) group and a non-systolic-blood-pressure low (NSBPL) group based on whether the baseline SBP was less than 120 mmHg (Table 3). SBP before and after treatment ranged from 109 mmHg to 105 mmHg (*p* = 0.109) in the SBPL group and from 134 mmHg to 125 mmHg (*p* < 0.001) in the NSBPL group. Only one patient could not tolerate even a 2.5 mg dose of vericiguat and developed symptomatic hypotension during follow-up. Besides, two patients developed gastrointestinal symptoms with abdominal distention as the main manifestation that could be relieved by Domperidone.

**Table 2:** The safety and effiency datas compared between baseline and follow-up.

Parameter	Baseline	Follow-up	*P*
Safety analysis			
Systolic blood pressure	120 (110, 139)	110 (104, 130)	<0.001
Creatinine	78.65 (64.63, 100.06)	76.72 (65.55, 94.61)	0.549
eGFR	86.56 (70.30, 99.01)	85.21 (69.54, 98.75)	0.521
Potassium	4.3 (4.0, 4.6)	4.3 (4.2, 4.7)	0.070
Efficacy analysis			
LVEF	36.0 (29.0, 45.0)	49.3 (36.7, 58.3)	<0.001
NT-proBNP	3,335 (944, 8,905)	1,270 (445, 1,980)	<0.001
Left atrium diameter	42.5 (37.5, 49.3)	41.9 (37.5, 45.4)	0.044
Left ventricular diameter	56.0 (48.2, 64.1)	55.4 (47.1, 62.4)	0.383
Mitral regurgitation area	4.1 (1.2, 8.0)	2.5 (0.0, 5.3)	0.013

eGFR, estimated glomerular filtration rate; LVEF, left ventricular ejection fraction; NT-proBNP, N-terminal pro-brain natriuretic peptide.

**Table 3:** Subgroup analysis based on blood pressure and renal function in a single-arm study.

Parameter	Baseline	Follow-up	*P*
Systolic blood pressure			<0.001*
<120 mmHg	109 (102, 114)	105 (90, 110)	0.109
≥120 mmHg	134 (125, 152)	125 (110, 135)	<0.001
eGFR			0.025*
<90 ml/min/1.73 m2	74.43 (57.97, 83.93)	73.18 (61.32, 85.24)	0.162
≥90 ml/min/1.73 m2	100.86 (95.61, 105.86)	96.53 (91.82, 109.87)	0.080

eGFR, Estimated glomerular filtration rate.

* Results of the comparison of the differences between the two groups.

Serum creatinine and eGFR remained unchanged before and after vericiguat treatment (*p* = 0.549 and 0.521, respectively). Meanwhile, no significant change was found in serum potassium (*p* = 0.070). Next, patients were divided into the renal insufficiency group (eGFR < 90 ml/min/1.73 m2) and the normal renal function group (eGFR ≥ 90 ml/min/1.73 m2) based on the baseline eGFR (Table 3). No significant change in eGFR was observed between patients with or without renal insufficiency (*p* = 0.162 and 0.080, respectively). However, the change in eGFR before and after treatment significantly differed between the two groups, with the renal insufficiency group showing a slower downward trend in eGFR after treatment (*p* = 0.025). Unfortunately, cardiovascular death occurred in six patients. The median time of death was 24 (6, 45) days.

Subsequently, we excluded populations with HFmrEF and HFpEF, and focused our safety analysis solely on individuals with HFrEF. The results demonstrated a significant reduction in systolic blood pressure, which decreased from 121 mmHg to 113 mmHg after treatment (*p* = 0.001). Renal function showed improvement post-treatment, as the eGFR increased from 79.13 ml/min/1.73 m² to 84.68 ml/min/1.73 m², with a significant difference observed before and after treatment (*p* = 0.015). Serum potassium levels, however, showed no significant change following treatment (*p* = 0.197).

### 3.3 Efficacy analysis

The efficacy analysis is detailed in Table 2. After receiving vericiguat for 68 days as the median, median LVD values ranged from 56.0 mm to 55.4 mm, with no statistical significance (*p* = 0.383). However, the median LVEF increased significantly from 36.0% to 49.3% (*p* < 0.001). Meanwhile, the median LAD reduced significantly from 42.5 mm to 41.9 mm (*p* = 0.044). Median MRA partially increased from 4.1 cm2 to 2.5 cm2 (*p* = 0.013). For laboratory data, 43 patients had baseline and follow-up NT-proBNP data, with median NT-proBNP values ranging from 3,335 pg/ml to 1,270 pg/ml (*p* < 0.001).

Subsequently, we conducted an efficacy analysis exclusively within the HFrEF population. The results indicated that, similar to the overall heart failure cohort, there were significant differences in LVEF (*p* < 0.001), LAD (*p* = 0.010), MR (*p* = 0.025), and NT-proBNP (*p* = 0.004) before and after treatment. However, no significant difference was observed in LVD before and after treatment (*p* = 0.958).

### 3.4 Subgroup analysis

The therapeutic efficacy of heart failure in different subgroups and specific populations remains unclear, including in women, the elderly, individuals with chronic kidney disease, and those with concomitant atrial fibrillation (13). Subsequently, subgroup analyses were conducted based on patient gender, age, renal function, and the presence of atrial fibrillation (AF) (Table 4).

**Table 4:** Subgroup analysis based on gender, age, renal function and atrial fibrillation in a single-arm study.

Parameter	LVEF			NT-proBNP		
	Baseline	Follow-up	*P*	Baseline	Follow-up	*P*
Gender			0.442*			0.557*
Man	35.1 (29.1, 45.0)	45.9 (36.1, 57.0)	<0.001	4,005 (761, 8,448)	1,290 (494, 2,110)	0.001
Woman	42.6 (29.0, 46.5)	54.7 (47.5, 61.0)	0.001	2,775 (1,780, 15,325)	1,210 (343, 1,890)	0.060
Age			0.862*			0.720*
> 65 years	43.2 (29.7, 49.1)	53.1 (36.5, 59.0)	<0.001	6,500 (846, 17,750)	1,310 (583, 8,410)	0.048
≤65 years	34.9 (29.0, 44.1)	47.0 (36.7, 57.0)	<0.001	2,860 (1,080, 7,090)	1,245 (431, 1,890)	0.001
Renal function (eGFR)			0.582*			0.859*
< 90 ml/min/1.73 m2	34.8 (28.0, 44.3)	46.5 (36.6, 56.8)	<0.001	5,815 (2,260, 9,943)	1,420 (426, 7,260)	0.018
≥90 ml/min/1.73 m2	42.5 (30.0, 49.0)	50.2 (36.8, 61.5)	<0.001	1,980 (319, 7,248)	1,052 (456, 1,308)	0.003
Atrial fibrillation			0.449*			0.282*
With atrial fibrillation	32.2 (23.8, 40.3)	40.3 (28.0, 47.2)	0.055	4,945 (1,458, 14,945)	3,100 (656, 7,835)	0.267
Without atrial fibrillation	37.5 (29.6, 45.3)	50.2 (37.3, 59.3)	<0.001	3,335 (887, 8,503)	1,250 (440, 1,960)	<0.001

eGFR, estimated glomerular filtration rate.

* Results of the comparison of the differences between the two groups.

In terms of gender differences, LVEF improved significantly after treatment in both male and female patients. However, no significant difference in NT-proBNP levels was observed in female patients post-treatment (*p* = 0.060). The change in LVEF and NT-proBNP between the two groups showed no significant difference.

Regarding the population with or without AF, although no significant differences were observed in the changes of LVEF and NT-proBNP between the two groups, in the AF subgroup, the changes in both LVEF and NT-proBNP post-treatment showed no statistically significant difference (*p* = 0.449 and 0.282, respectively).

No significant differences in treatment outcomes were observed across different age groups (with a cutoff age of 65 years) or renal function categories (with an eGFR cutoff of 90 ml/min/1.73 m²).

### 3.5 PSM

For vericiguat safety and efficacy evaluation, the PSM method was used to create an external control for the vericiguat group. The real-world IPD of 75 HF subjects from the study hospital's electronic health records were collected. To balance the baseline characteristics between the vericiguat group and the external control group, the SMD for each covariate of baseline characteristics were calculated, resulting in two PSM-matched groups, with 63 participants in the vericiguat group and 63 subjects in the external control group (Figure 1). Detailed subject characteristics and a comparison of baseline characteristics between the vericiguat group and the control group are shown separately after the PSM matching in Table 5.

**Table 5:** Characteristics of the patients in PSM at baseline.

Parameter	Total *n* = 126	Vericiguat Group *n* = 63	Control Group *n* = 63	*P*
Basic characteristics				
Age	65 (58, 70)	65 (58, 72)	65 (56, 69)	0.742
Male gender	91 (72.2%)	47 (74.6%)	44 (69.8%)	0.551
Systolic blood pressure	127 (111, 145)	120 (109, 135)	135 (117, 156)	0.003
Comorbidities				
Hypertension	75 (59.5%)	33 (52.4%)	42 (66.7%)	0.102
Diabetes mellitus	34 (27.0%)	13 (20.6%)	21 (33.3%)	0.108
Atrial fibrillation	11 (8.7%)	8 (12.7%)	3 (4.8%)	0.115
After coronary stent	27 (21.4%)	16 (25.4%)	11 (17.5%)	0.278
Etiology				
Ischemic cardiomyopathy	110 (87.3%)	55 (87.3%)	55 (87.3%)	1.000
Nonischemic cardiomyopathy	16 (12.7%)	8 (12.7%)	8 (12.7%)	1.000
Laboratory data				
Creatinine	78.70 (63.90, 97.65)	77.56 (64.80, 96.74)	81.25 (61.73, 99.00)	0.596
eGFR	84.57 (66.85, 97.51)	86.75 (70.29, 98.85)	82.95 (64.88, 97.02)	0.456
Potassium	4.3 (4.0, 4.6)	4.2 (4.0, 4.5)	4.3 (3.9, 4.6)	0.506
NT-proBNP	2,620 (912, 7,210)	2,800 (883, 9,030)	2,500 (934, 5,510)	0.433
Echocardiography				
LVEF	38.5 (31.7, 44.5)	38.0 (29.4, 46.0)	38.5 (33.6, 43.0)	0.838
Left atrium diameter	42.1 (38.0, 47.5)	41.9 (37.6, 49.0)	42.4 (38.6, 45.1)	0.572
Left ventricular diameter	54.2 (48.8, 62.3)	55.0 (47.1, 64.1)	54.2 (49.8, 62.2)	0.757
Mitral regurgitation area	4.1 (1.7, 7.2)	4.1 (1.0, 8.4)	3.8 (1.7, 6.5)	0.982

eGFR, estimated glomerular filtration rate; NT-proBNP, N-terminal pro-brain natriuretic peptide; LVEF, left ventricular ejection fractions.

In terms of effectiveness evaluation, LVEF and NT-proBNP were significantly improved before and after treatment in both vericiguat group and external control group. However, there was no significant difference between the two groups (*p* = 0.367 and 0.543, respectively). Although LAD and MRA Improved in the control group but not in the vericiguat group, this may be due to the shorter follow-up period.

In terms of safety evaluation, the renal function of the vericiguat group did not change significantly before and after treatment, while the renal function of the control group worsened after treatment. eGFR was significantly different between the two groups (*p* = 0.024), and the deterioration was more significant in the control group. Although potassium varied significantly before and after treatment in the vericiguat group (*p* = 0.021), they almost all fluctuated within the normal range. The results of the case-control study are presented in Table 6.

**Table 6:** The safety and effiency datas compared between baseline and follow-up in PSM.

Parameter	Vericiguat group			Control group			ΔP
	Baseline	Follow-up	*P*	Baseline	Follow-up	*P*	
Safety analysis							
Creatinine	77.56 (64.80, 96.74)	76.99 (66.05, 94.99)	0.997	81.25 (61.73, 99.00)	92.80 (72.69, 104.47)	0.006	0.077
eGFR	86.75 (70.29, 98.85)	83.93 (68.81, 95.89)	0.945	82.95 (64.88, 97.02)	72.55 (59.02, 90.56)	0.001	0.024
Potassium	4.2 (4.0, 4.5)	4.3 (4.2, 4.7)	0.021	4.3 (3.9, 4.6)	4.4 (4.1, 4.7)	0.049	0.857
Efficacy analysis							
Left ventricular ejection fraction	38.0 (29.4, 46.0)	49.0 (36.5, 58.0)	<0.001	38.5 (33.6, 43.0)	46.0 (37.0, 55.0)	<0.001	0.367
NT-proBNP	2,800 (883, 9,030)	1,300 (539, 2,400)	0.002	2,500 (934, 5,510)	1,020 (354, 7,930)	0.033	0.543
Left atrium diameter	41.9 (37.6, 49.0)	41.8 (37.5, 45.4)	0.160	42.4 (38.6, 45.1)	40.0 (38.0, 43.5)	0.015	0.442
Left ventricular diameter	55.0 (47.1, 64.1)	55.0 (46.9, 63.4)	0.913	54.2 (49.8, 62.2)	55.0 (48.6, 59.2)	0.064	0.250
Mitral regurgitation area	4.1 (1.0, 8.4)	2.8 (1.3, 5.6)	0.216	3.8 (1.7, 6.5)	2.0 (0.0, 4.7)	<0.001	0.079

eGFR, estimated glomerular filtration rate; NT-proBNP, N-terminal pro-brain natriuretic peptide.

## 4 Discussion

The present study initially evaluated the safety and efficacy of vericiguat therapy in patients with acute HF in real-world practice. A partial subgroup analysis and PSM was also performed to better evaluate its role.

### 4.1 Safety analysis

Symptomatic hypotension is a relatively common adverse effect of vericiguat, which is due to its vasodilatory effect. The VICTORIA trial found that the incidence of symptomatic hypotension and syncope was higher in the vericiguat group compared to the placebo group, but with no statistically significant difference (3). Subgroup analysis of the VICTORIA trial revealed that patients older than 75 years and those receiving ARNI treatment had a slight initial decrease in systolic blood pressure (SBP), which then returned to baseline levels. Additionally, the study found that patients in both the vericiguat and control groups with a baseline SBP <110 mmHg showed an increasing trend in SBP over time (14). This study also noted that although there was a significant decrease in SBP in individual treatment groups before and after treatment, this could be due to the concurrent use of other antihypertensive heart failure medications, such as ARNI and β-blockers. However, subgroup analysis in this study showed no significant change in blood pressure in the SBPL subgroup before and after treatment, which further supports the feasibility and safety of vericiguat treatment in hypotensive patients.

The use of angiotensin-converting enzyme inhibitors, β-blockers, spironolactone and other drugs is restricted or even discontinued in patients with chronic HF due to increased creatinine levels, renal dysfunction and other reasons. All these may have adverse effects on the prognosis and condition of HF patients. The VICTORIA subgroup trial examined the relationship between the efficacy of vericiguat therapy and renal function in HFrEF patients and found that the trajectories in eGFR and creatinine with vericiguat therapy were similar to those with placebo (15). Our data showed that baseline creatinine and eGFR were 80.69 μmol/L and 84.27 ml/min/1.73 m2 respectively and remained unchanged during the vericiguat treatment. Similar observations were reported in the VICTORIA trial. Additionally, we found that eGFR did not change significantly after treatment regardless of renal insufficiency. However, a statistically significant difference in the change in eGFR was found between the two groups before and after treatment, with the renal insufficiency group exhibiting a more gradual deterioration trend after treatment (*p* = 0.025). In a further case-control study, we found that eGFR in the vericiguat group did not change significantly before and after treatment (*p* = 0.719), while eGFR in the external control group decreased significantly after treatment (*p* = 0.001), which was statistically significant (*p* = 0.024).

In recent years, several preclinical studies have suggested that sGC stimulators possess nephroprotective effects. Atteia et al. demonstrated that the long-term treatment with the sGC stimulator Iisiquiritigenin effectively and dose-dependently improved chronic renal dysfunction and endothelial dysfunction induced by adenine. This intervention not only alleviated renal fibrosis and apoptotic markers but also reduced aortic calcification (16). Lichtenberger et al. proposed that sGC activator therapy could enhance cGMP concentrations in tissues, leading to the dilation of renal microvasculature, improved blood flow, and enhanced oxygenation. This intervention significantly mitigated the reduction in renal weight, cellular damage, fibrosis, and inflammation (17). This suggested that vericiguat therapy may have a potential renoprotective mechanism, which qualifies its potential application in HF patients with renal insufficiency.

In addition, a previous study reported that vericiguat therapy neither increased nor decreased serum potassium levels and therefore, it may be safely administered to patients with elevated serum potassium concentrations (15), which is consistent with our conclusions.

### 4.2 Efficacy analysis

The single-arm study found significant improvements in NT-proBNP, LVEF, LAD and MRA. The lack of significant changes in LVD might be due to short follow-up time or small sample size. In case-control studies, LVEF and NT-proBNP showed significant improvement after treatment in both groups. The VICTORIA subgroup trial indicated that vericiguat significantly decreased NT-proBNP levels in patients with worsening HFrEF compared with the placebo (18). This is an initial indication of the efficacy of vericiguat therapy in patients hospitalized for HF. While this was true for other drugs for patients with HF, vericiguat played a crucial role. However, large randomized controlled trials, with longer follow-up time are needed to further confirm the effectiveness of vericiguat.

In patients with HFrEF, the left atrium typically undergoes dilation due to impaired left ventricular pumping function, resulting in eccentric remodeling. Additionally, left atrial strain has been shown to be a strong predictor of recovery of ejection fraction in HFrEF patients, independent of left ventricular strain. Vericiguat, by increasing cGMP levels, can improve hemodynamic status, reduce peripheral vascular resistance, decrease the cardiac afterload, and promote vasodilation. In this study, although a significant reduction in LAD was observed, there was no significant change in LVD. This may be attributed to the fact that the effects of vericiguat on the left ventricle primarily involve improving ventricular diastolic function and hemodynamic status, rather than directly altering its anatomical structure. It can be hypothesized that vericiguat's effects on cardiac chamber size may differ, as it significantly reduces the pressure burden on the left atrium, leading to notable improvements in left atrial size. In contrast, the impact on the left ventricle primarily manifests as functional improvement, which may require a longer duration or additional therapeutic interventions before structural changes in the left ventricle can be observed.

### 4.3 Subgroup analysis

In gender, ischemic heart disease risk in women includes biomedical, behavioral, and psychosocial contributors (19). The study found that the prevalence of heart failure is higher in women over the age of 80 compared to men (20). Similarly, in large-scale trials of heart failure with reduced ejection fraction (HFrEF), women were older than men and exhibited more symptoms along with poorer overall health status (21). A post analysis showed that elderly, postmenopausal women with HFpEF might benefit from nitric oxide-sensitive guanylyl cyclase stimulation (22). This is because comorbidities in conjunction with estradiol decline in the postmenopausal age might impair cyclic guanosine monophosphate signaling to a higher extent in female patients (23). Our research found no significant differences in the changes of left ventricular ejection fraction (LVEF) and NT-proBNP between genders (*p* = 0.442 and 0.557, respectively), which is consistent with the results of the VICTORIA trial (24). Such a difference in mechanism may be difficult to be manifested in clinical setting either.

AF is one of the most common complications of HF. The mechanisms by which HF contributes to the development of AF are multifactorial, involving factors such as neurohormonal activation, cardiac stretch, oxidative stress, and endothelial dysfunction, all of which contribute to the inflammatory state characteristic of HF (25, 26). Chronic inflammation in HF patients is marked by elevated levels of pro-inflammatory molecules, which trigger atrial fibrosis, enlargement, and cell death, thereby disrupting the normal structure and function of atrial tissue and facilitating the development and maintenance of AF (27). Previous studies have suggested that the positive effect of vericiguat on the primary composite outcome and its components remains consistent regardless of the presence of atrial fibrillation at baseline (28), which is consistent with the findings of our study. However, animal studies have shown that vericiguat can reverse atrial enlargement, reduce myocardial fibrosis, prevent the shortening of atrial effective refractory periods, and decrease the inducibility of AF, thereby significantly improving the structural and electrophysiological remodeling associated with AF (29). These findings suggest the potential of vericiguat as a therapeutic agent for AF, warranting further investigation into its effects and significance in the treatment of AF in future studies.

### 4.4 Dose selection in real world

The SOCRATES-REDUCED Randomized Trial reported a dose-effect relationship, in which higher vericiguat doses were associated with greater reductions in NT-proBNP levels. Meanwhile, mortality and HF hospitalization rates also tended to decrease with higher vericiguat doses (30). The SOCRATES-PRESERVED phase IIb study found that vericiguat at a dose of 10 mg improved the quality of life in patients with HFpEF (31). Thus, 10 mg is conventionally recommended as a target dose of vericiguat; however, only 17.6% of patients achieved the target dose in our study. Most of our patients selected the 5 mg dose largely out of choice rather than adverse drug reaction. Therefore, future studies should pay close attention to the up-titration of vericiguat up to the maximum dose by more propaganda and education.

### 4.5 Study limitations

This study is a single-center retrospective study with a very small sample size, with a short follow-up period of only 68 days as the median. Due to the small sample size, some clinically significant differences could not reach statistical significance. There were 20 patients who did not complete outpatient follow-up for personal reasons, which may have increased selection bias. Furthermore, our study did not differentiate between patients with *de novo* acute HF or decompensation of chronic HF, nor did it distinguish between different HF subtypes. This introduces an additional level of uncertainty to the study results.

Previous clinical studies have mostly included patients with chronic heart failure with HFrEF or HFpEF. For example, the VICTORIA trial (3), the VITALITY-HFpEF Randomized Clinical Trial (32), the SOCRATES-REDUCED Randomized Trial (30), the SOCRATES-PRESERVED study (31) and some subgroup studies of the VICTORIA study. Several Phase Ib studies have considered the use of vericiguat for the prevention and treatment of angina pectoris in patients with chronic coronary syndromes (33, 34). Our study innovated the use of vericiguat in patients with acute decompensated heart failure and found that vericiguat is safe and contributes to the improvement of symptoms and cardiac function in these patients.

In summary, the current study demonstrated that vericiguat therapy is safe, feasible and effective in patients with acute HF. Moreover, vericiguat had a better protective effect on blood pressure and renal function than other anti-HF drugs. Vericiguat may also have a unique protective mechanism for the kidneys. Nonetheless, large randomized controlled trials are warranted to further validate these conclusions.

## Data Availability

The raw data supporting the conclusions of this article will be made available by the authors, without undue reservation.
